# Quality of Life, Anxiety, and Depression in Caregivers of Community-Dwelling Heart Failure Patients

**DOI:** 10.3390/healthcare13161986

**Published:** 2025-08-13

**Authors:** Maria Polikandrioti, Athanasia Tsami, Vasiliki Tsoulou, Andriana Maggita

**Affiliations:** Department of Nursing, University of West Attica, 12244 Athens, Greece

**Keywords:** quality of life, SF36, heart failure, anxiety, depression

## Abstract

**Background/Objectives:** Patients with heart failure (HF) experience increased morbidity, limited daily activities, and diminished quality of life (QoL), thus relying on a family member, widely known as informal caregiver, for support. The objective of this study was to explore (a) QoL, anxiety, and depression; (b) factors associated with QoL; and (c) the impact of associated factors on QoL among HF caregivers. **Materials and methods:** Data collection was performed using the *36*-Item Short Form *Survey* (*SF-36*), the Hospital Anxiety and Depression Scale (HADs), and the European Heart Failure Self-care Behavior Scale (EHFScBS). Also recorded were characteristics of caregivers and patients. **Results:** In the present study, 110 HF caregivers and the family members they provided care to were enrolled. The majority of caregivers were patients’ spouses (60%) and were female (71.8%). Within a QoL score range of 0–100, caregivers showed moderate to high levels in role-physical, role-emotional, emotional well-being, and pain (median: 75, 66.7, 64, and 67.5, respectively); moderate QoL levels in energy/fatigue, social functioning, and general health (median: 55, 56.3, and 62, respectively); and poor QoL levels in physical functioning (median: 18). Moreover, 64.5% of caregivers had anxiety and 41.8% had depression. Caregivers with HADs scores that indicate anxiety and depression had worse QoL (*p* = 0.001). No association was detected between caregivers’ QoL and patients’ HADs and self-care. **Conclusions:** QoL and anxiety/depression merit further research by clinicians, health systems, and policymakers so that evidence-based policies and interventional programs tailored to their needs can be implemented.

## 1. Introduction

The progressive clinical syndrome of heart failure (HF) is growing dramatically worldwide, mainly due to the availability of advanced diagnostic tools and medical treatments [1]. According to estimates, in 2019, the prevalence of HF was 17 per 1000 persons across 13 European countries, ranging from ≤12 in Greece and Spain to >30 per 1000 individuals in Lithuania and Germany [2]. HF is the leading cause of hospitalization and re-admissions among older adults [3,4,5]; furthermore, it is associated with increased mortality, since 50% of patients will pass away within 5 years of diagnosis [3]. Regarding stable and ambulatory HF patients, European data demonstrate a 7% and 32% rate of 12-month all-cause mortality and 12-month hospitalization, respectively [5]. Approximately 75% of patients with HF have difficulties in performing their daily activities [4].

The adverse impact of HF is also expanded to family members who perceive the role of informal caregiver as a duty or responsibility. HF caregivers lead a long-term journey with several changes or transitions in the context of care [4,6]. More specifically, tasks provided by HF caregivers are categorized into direct (hands-on) tasks, such as monitoring vital signs or weighing the patient, and indirect ones (hands-off), such as support in navigating the health care system [7]. It is not rare for HF caregivers to report unfulfilled needs, inadequate social support networks, insufficient caregiving knowledge or competence, low preparedness for care at home, diminished communication with health care professionals, and social isolation [4,6,7]. More strikingly, HF caregivers experience a gap between the expectations of life and the prevailing reality [6]. Frequently, a remarkable imbalance is observed between the demands caregivers receive and their ability to cope with these demands [8]. In contemporary times, the caregiving role has proved to be complex due to advances in HF treatments, either medical or surgical. Furthermore, caregivers’ comprehension of HF has an impact on patients’ treatment decisions [9]. The primary stressors (care tasks) and the secondary ones (role strain, loss of identity) exert a negative impact on caregivers’ well-being [10].

Indeed, all aforementioned factors, along with the unpredictable nature of this clinical syndrome and the demanding daily management tasks, may adversely affect caregiver’s health outcomes, including quality of life (QoL) and mental health [4,11]. HF caregivers spend a great deal of time providing support to patients, which disrupts their daily life, social interactions, and work productivity; thus, they experience psychological disturbance [4]. In terms of mental health, depressive symptoms are common among HF caregivers, ranging from 6% to 64% [12]. Despite the fact that caregiving is a rewarding activity, caregivers experience depression as a sequela of their role, which predicts levels of QoL [13].

In longitudinal HF care, the role of caregivers is an arduous challenge, and clinicians, researchers, health systems, and policymakers strive to accelerate further development of HF caregiving science [1]. Data on HF caregiver outcomes such as QoL are not extensive, but considerable progress has been noticed during recent years.

With the developing attention on moving HF management towards outpatient settings and minimizing the burden upon hospital medical resources, it is obvious that assessment of anxiety/depression and QoL among HF caregivers gains a rising importance in the realm of care. In the community, the role of HF caregivers is of utmost importance in preventing hospitalization and avoiding decompensation. Given that caregiving has beneficial effects on the physical and psychological health of both the patient and caregiver, the need to explore and improve HF caregiver outcomes has emerged to the forefront of clinical practice.

In an attempt to address all these issues and fill the deficits in the knowledge about caregiver-centered decisions, this study aimed to explore (a) QoL, anxiety, and depression among HF caregivers of community-dwelling patients; (b) patients’ and caregivers’ characteristics associated with caregivers’ QoL; (c) patients’ self-care, anxiety, and depression; and (d) the impact of patients’ and caregivers’ characteristics on caregivers’ QoL.

## 2. Materials and Methods

### 2.1. Design, Setting, and Period of the Study

In this cross-sectional study, 110 HF caregivers and 110 patients they provided care to were enrolled. HF caregivers accompanied their patients to regular monitoring and follow-up in an outpatient clinic of a public hospital in Attica from December 2023 to January 2024. Participants were selected by the method of convenience sampling. According to this method, participants are selected based on proximity and ease of access and not through a random or systematic selection process (non-probability sampling method) [14].

### 2.2. Inclusion and Exclusion Criteria of the Sample

In this study, only informal family caregivers who provided care to stable community-dwelling patients with a diagnosis of HF and no prior hospitalization within the last 6 months were included.

According to European Society of Cardiology (ESC) guidelines, chronic, stable HF patients are those having no or mild signs and symptoms which have not changed for at least one month [5]. Criteria for inclusion in this study for both patients and caregivers were (a) understanding the Greek language and (b) the ability to comprehend, read, and sign the informed consent form. Exclusion criteria for HF caregivers and patients were (a) being diagnosed with a psychiatric illness and (b) receiving medical treatment for psychiatric disorder.

### 2.3. Data Collection and Procedure

Data was collected after the end of outpatient monitoring and follow-up care by the method of an interview in an office at the outpatient clinic. The procedure lasted approximately 15 min for each patient.

### 2.4. Research Instrument

Data collection was performed using the *36*-Item Short Form *Survey* (*SF*-*36*), the Hospital Anxiety and Depression Scale (HADs), and the European Heart Failure Self-care Behavior Scale (EHFScBS). The data collected for each patient and caregiver included demographic characteristics and other self-reported characteristics. These variables were selected through clinical observations and a literature review [4,7,9,11].

#### 2.4.1. Measurement of Caregivers’ QoL (SF36)

The *36*-Item Short Form *Survey* (*SF*-*36*) scale was used to assess caregivers’ QoL. This scale (created by Ware and colleagues in 1993) assesses physical and mental health. It consists of 36 questions comprising 8 dimensions: physical functioning, role-physical, physical pain, general health, energy/fatigue, social functioning, role-emotional, and emotional well-being. Respondents answer the questions on Likert-type scales. The scores assigned to the questions are summed separately for questions that evaluate the 8 dimensions. Higher scores indicate better QoL. In addition, Physical (*PCS*) and Mental (MCS) Component Summary scores of caregivers were obtained, and they were compared with the average score of the general population. The scores for each QoL component range from 0 to 100, with higher scores indicating better QoL. The SF-36 is a valid and reliable measure used in multiple populations, including HF patients and caregivers [15,16]. The scale was tested for its validity and reliability in the Greek population by Pappa et al. (2005) [15].

#### 2.4.2. Measurement of Anxiety and Depression (HADs)

For the evaluation of the mental health (depression and anxiety) of caregivers, as well as their recipients of care, the “Hospital Anxiety and Depression Scale (HADs)” was used. This scale was proposed in 1983 by Zigmond AS & Snaith RP. The scale consists of 14 questions that assess how respondents felt during the previous week. Respondents are able to answer every question on a 4-point Likert scale from 0 to 3. Seven of the fourteen questions assess the level of depression and the other seven evaluate the anxiety level. Scores attributed to questions are summed separately for anxiety and depression, leading to two scores with a range of 0–21. Higher scores indicate higher levels of anxiety and depression. In addition, the following categorization is widely used in the literature: a score of 0–8 indicates no anxiety or depression, and a score above 8 indicates anxiety or depression [17,18]. This scale has been tested for its validity and reliability in the Greek population by Μichopoulos et al. (2008) [18].

#### 2.4.3. Measurement of Patients’ Self-Care

The European Heart Failure Self-care Behavior Scale (EHFScBS) is a validated tool designed to assess self-care behaviors in HF patients. It evaluates key aspects of daily self-management, including medication adherence, fluid and salt intake, symptom monitoring, and help-seeking behavior. The scale consists of 9 items rated on a Likert scale (1–5). The total score ranges from 9 to 45, with lower scores indicating better self-care behavior. This scale was validated by Lambrinou et al. in 2014 in a sample of 128 HF patients, NYHA class I-IV, from the cardiology units and outpatient departments of four large public hospitals in Cyprus. The Cronbach alpha of the 9 items ranged from 0.561 to 0.645. The same researchers reported comparable Cronbach’s alpha values in previous measurements in a UK population sample [19].

### 2.5. Ethical Considerations

The present study was approved by the Research Committee of the public hospital (number 105/22-2-2021). Participants were informed of the purposes of the study by the researcher and their written informed consent was obtained. Data collection guaranteed anonymity and confidentiality. All subjects were informed of their rights to refuse or discontinue participation in the study according to the ethical standards of the Declaration of Helsinki (1989) of the World Medical Association.

### 2.6. Statistical Analysis

Categorical data are presented as absolute and relative frequencies (%), while continuous data are presented as means, standard deviations, medians, and interquartile ranges. Normality of the data was tested with the Kolmogorov–Smirnov criterion and graphically with Q-Qplots and Histograms. Non-parametric tests, namely the Mann–Whitney and Kruskal–Wallis tests, as well as Spearman’s rho correlation coefficient, were used to test for associations between caregivers’ QoL, participants’ characteristics, and patients’ self-care as well as anxiety and depression. Cronbach’s a indexes were calculated to assess the internal consistency of participants’ answers. Values above 0.7 indicate consistency.

Multiple linear regression was performed to estimate the effect of all variables under evaluation on caregivers’ QoL. Results are presented as β regression coefficients and 95% confidence intervals (95% CIs). An observed level of 5% was considered statistically significant. All statistical analyses were performed with SPSS v.29 (SPSSInc, Chicago, IL, USA).

## 3. Results

### 3.1. Sample Description (Caregivers)

Table 1 presents a description of the caregiver sample. The majority of caregivers were the patients’ spouses (60%), female (71.8%), above 60 years old (31.8%), employed (57.3%), had completed secondary education (35.5%), were residents in Attica (58.2%), and had two children (36.4%). Regarding their self-reports about caregiving, the majority stated that they needed written information about HF patients’ self-care (87.2%), needed to learn new skills (75.0%), always supported the patients in daily management tasks (38.2%), often neglected their own health (40.0%), and often participated in medical decision making (55.5%).

### 3.2. Sample Description (Patients)

The majority of the patients were male (66.4%), >70 years old (55.5%), NYHA III (43.6%), had primary education (50.9%), were pensioners (62.8%), were residents of Attica (54.5%), and reported that they needed written information on HF (70.9%) and to learn new skills (58.2%) (Table 2).

### 3.3. Caregivers’ QoL

**Figure 1:**
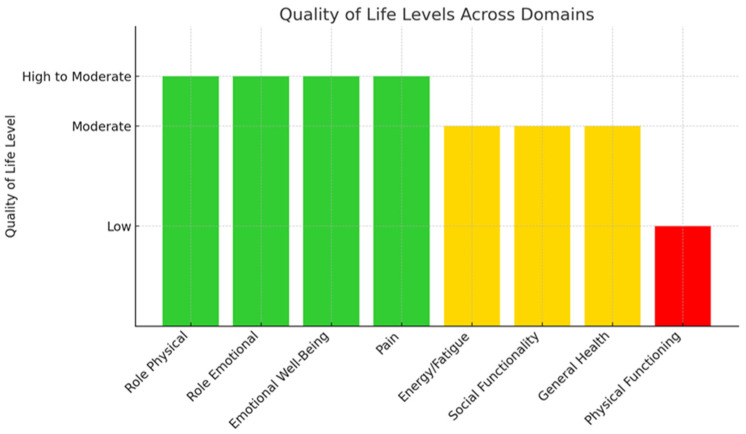
Quality of life level.

Table 3 presents the results regarding caregivers’ QoL. Caregivers showed moderate to high QoL levels in role-physical, role-emotional, emotional well-being, and pain (median: 75, 66.7, 64, and 67.5, respectively) and moderate QoL levels in energy/fatigue, social functioning, and general health (median: 55, 56.3, and 62, respectively). Poor QoL was observed in physical functioning (median: 18). Furthermore, the Physical (*PCS*) and Mental (MCS) Component Summary scores of caregivers indicated a worse than average QoL compared to the general population (medians: 35.9 and 47.5—less than 50). Their physical status was worse than the mental one. All Cronbach’s values were above 0,7, indicating high internal consistency in answers of participants (Figure 1).

### 3.4. Anxiety/Depression Among HF Caregivers and Their Patients

Regarding anxiety/depression among patients and their caregivers, Table 4 shows that 64.5% and 41.8% of caregivers had anxiety and depression, respectively, while 56.4% and 59.1% of patients had anxiety and depression, respectively, with a cut-off score of 8. All Cronbach’s values were above 0.8, indicating high internal consistency in participants’ answers.

### 3.5. Patient’s Self-Care Behavior

Table 5 presents the results regarding patients’ self-care behavior. Half of the patients scored below 17.5 (median). Moreover, half of the patients scored between 14 and 23. This result indicates that patients had moderate levels of self-care. Cronbach’s a value was 0.775, indicating high internal consistency in participants’ answers.

### 3.6. Caregiver-Related Factors Associated with Caregivers’ QoL

Table 6 presents the associations between the characteristics of HF patients’ caregivers and their QoL. The Physical (*PCS*) and Mental (MCS) Component Summary scores of caregivers’ QoL were found to be statistically significantly associated with the type of relationship to the patient (*p* = 0.001 and *p* = 0.003, respectively), gender (*p* = 0.001 and *p* = 0.012, respectively), age (*p* = 0.001 and *p* = 0.001, respectively), residency (*p* = 0.004 and *p* = 0.010, respectively), number of children (*p* = 0.037 and *p* = 0.002, respectively), level of self-reported information about the self-care of the patients they care for (*p* = 0.001 and *p* = 0.001, respectively), the need to learn new skills (*p* = 0.005 and *p* = 0.001, respectively), the provision of support to perform daily management tasks (*p* = 0.001 and *p* = 0.001, respectively), neglect of their own health (*p* = 0.001 and *p* = 0.001, respectively), and their participation in medical decisions (*p* = 0.001 and *p* = 0.001, respectively). Specifically, caregivers who were spouses of patients had statistically significantly worse PCS and MCS scores (medians: 33.5 and 38.8). Caregivers under 60 years of age had better *PCS* (median <40 years: 39; median 41–50 years: 40.3; median 51–60 years: 36.2) compared to caregivers over 60 years of age (median 29.3) as well as better MCS (median < 40 years: 63.6; median 41–50 years: 49.3; median 51–60 years: 51.8) compared to caregivers over 60 years of age (median: 32.8). In addition, caregivers who lived in the province (medians: 30.4 and 35.1), those who had more than two children (median: 32.1 and 40.8), those who reported to be “little” informed about the patient’s self-care (medians: 28.9 and 32.3), those who declared the need to learn new skills (medians: 34.63 and 43.1), and those who declared that they always provided support in daily management tasks (medians: 30.7 and 33.9), always neglected their own health (medians: 31.2 and 32.6), and always participated in decision making (medians: 29.1 and 31.9) had significantly worse *PCS* and MCS scores.

Regarding the association between HADs and caregivers’ QoL, it was found that caregivers with HADs scores that indicate anxiety and depression had worse QoL in terms of PCS and MCS, with medians for anxiety of 32 and 37.3 vs. 38.9 and 59.8 and medians for depression of 30.6 and 33.2 vs. 39 and 57.3, *p* = 0.001 for all.

### 3.7. Patient-Related Factors Associated with Caregivers’ QoL

Table 7 presents the associations between patients’ characteristics and caregivers’ QoL. The Physical Component Summary (PCS) score of caregivers was found to be statistically significantly associated with patients’ gender (*p* = 0.035). Specifically, caregivers of male patients had statistically significantly worse PCS (median 35.6) than caregivers of female patients (median 39.8). The Mental Component Summary (MCS) score of caregivers was found to be statistically significantly associated with patients’ occupation (*p* = 0.029). Specifically, caregivers of patients whose occupation was classified as “household” had a statistically significant worse MCS (median 34.1) than caregivers of patients who were employees (median 52.8) and pensioners (median 47.6). No association was detected between patient HADs and caregiver QoL. Likewise, no association was detected between patient’s self-care behavior and caregiver QoL.

### 3.8. Effect of Characteristics on Caregiver’s QoL

Multiple linear regression was performed to estimate the effect of independent factors/characteristics on caregiver’s QoL. (Table 8).

Caregivers who had depression on HADs assessment had worse PCS scores by 6.2 points than caregivers who did not (b = −6.22 95%CI: −9.7–−2.73, *p* = 0.001). Furthermore, caregivers who had anxiety or depression on HADs assessment had worse MCS scores by 9 and 8 points than caregivers who did not (b = −9.27 95%CI: −14.89–−3.68, *p* = 0.002 and b = −8.21 95%CI: −14.85–−1.57, *p* = 0.016, respectively).

Caregivers who “rarely” supported patients in managing daily tasks had better MCS scores by 7.9 points than caregivers who reported supporting them “often” (b = 7.90 95%CI: 0.49–15.30, *p* = 0.037).

Caregivers who declared that they participated in medical decision making “often or rarely” had better MCS scores by 12 and 14 points than caregivers who declared that they “always” participate in it (b = 12.19 95%CI: 3.23–21.14, *p* = 0.009 and b = 14.23 95%CI: 4.15–24.31, *p* = 0.006, respectively).

## 4. Discussion

The aim of the present study was to explore the QoL of caregivers of patients with stable HF living in the community with no prior hospitalization within the last 6 months. Also, the factors associated with QoL—a. caregivers’ and patients’ characteristics, b. patients’ self-care, and c. anxiety and depression in both caregivers and patients—were explored. In terms of HF patients’ caregivers, the results showed poor QoL in physical functioning and indicated that anxiety and depression affected their QoL. Regarding patients, their anxiety, depression, and self-care had no association with caregivers’ QoL.

The finding that HF caregivers had poor QoL in physical functioning is explained by several factors related to their role, such as short sleep duration, fatigue, and practical caregiving-related issues [4,9]. Similar studies in Greece [20,21] exploring the QoL of HF caregivers showed worse QoL in the Physical Component Summary [21], as well as in physical functioning, pain, and general health [20], but the main difference is that HF patients in those studies were hospitalized, whereas in the present one, they were community-dwelling, which means that caregivers provide the needed care to their loved person in the familiar environment of the home, thus maintaining a sense of normal life. Contrariwise, caregivers have 1.48 times higher odds of diminished QoL when HF patients use hospital services compared to when they do not [8].

According to the results, worse physical and mental QoL among caregivers was associated with their anxiety and depression. In Italy, Petruzzo et al. [22] demonstrated that depression in HF caregivers influenced both dimensions of QoL, while anxiety influenced only mental QoL. Chung et al. [23], in the USA, showed that HF caregivers with depressive symptoms had poor functional status, low perceived control, and high caregiving distress. In advanced HF patients, high anxiety levels were associated with worse QoL [24]. In brief, high anxiety and depression of HF caregivers may reflect that their life is restricted [22]. Provided that caregivers are the main source of help to patients, they should also receive attention and support from medical staff [25]. Unfortunately, health system barriers limit the assessment of the mental health for HF caregivers [26].

The present result that anxiety and depression in HF patients showed no association with caregivers’ QoL contradicts published data showing that HF caregivers’ psychological QoL and anxiety were associated with patients’ anxiety in clinical settings [25].

Moreover, in the present study, patients’ self-care had no association with HF caregivers’ QoL. This finding is not in line with a study conducted Kim et al. [27] demonstrating that low patient self-care was associated with the physical function of HF caregivers. Family caregivers have a fundamental role in encouraging patients to improve their self-care and frequently report satisfaction from caregiving. The positive aspects of caregiving and its intrinsic rewards may indirectly increase QoL [28]. In the current study, patients had moderate levels of self-care, as measured by the EHFScBS. This research instrument helps health care professionals to identify patients in need of targeted interventions which improve self-care and reduce hospital readmissions [19].

Spouse caregivers had worse QoL in physical and mental components. Spouses generally provide household help, personal care, and instrumental and emotional support. Nowadays, increased longevity elevates the potential for caregiving by spouses, which is supplementary to other types of caregiving, such as by professional care providers or by adult children. These supplementary types of caregiving are frequent when the health of the spouse is declining. Diminished spousal care may reflect macro-level changes, such as less family solidarity or public provision of home care. From a policy perspective, lack of spousal care necessitates publicly provided formal care [29].

In terms of residency, HF caregivers who lived in the province had worse QoL in physical and mental components. Residency may affect QoL indirectly. Rural residents are more likely to have limited access to health care services but simultaneously have expanded social networks, strong personal bonds, and a high reliance on family members. Consequently, caregivers have added responsibilities, being on the front line of providing support and care to HF recipients. Extending formal services and resources to rural areas might be a solution for improving family-centered care and QoL [10].

A noticeable finding is the worse physical and mental QoL among HF caregivers who had little information about patients’ self-care. This group of caregivers may feel unable or unprepared to manage with long-term HF care; thus, they may not actively participate in treatment [30]. Insufficient knowledge or low confidence in HF management and limited caregiving experience may increase anxiety and depression [22,25]. Educational and supportive interventions help caregivers to undertake responsibility and preserve the integrity and quality of their lives [30].

Also, worse physical and mental QoL was observed among HF caregivers who ignored their own health. Inevitably, caregivers prioritize patients’ needs and assume responsibilities regardless of their own physical ability or emotional burden [13]. On a daily basis, HF caregivers spend 5 to 8 hours or more providing care, thus being at a higher risk of physical burden or of neglecting their own self-care (e.g., exercise, healthy diet) [4,8]. HF caregivers spend, on average, 20 h/week on caregiving activities, which means above 3000 h over 3 years. The amount of time spent on HF caregiving weekly equates to more than half (53%) of the weekly working time (37 h) in Europe, indicating that HF caregiving is equivalent to a part-time occupation. Indeed, when HF caregivers invest a considerable amount of time providing care, negative outcomes, such as poor psychological well-being and lower QoL, in turn emerge to the surface [4].

Worse physical and mental QoL was reported by HF caregivers who declared the need to learn new skills, as well as those who always provided support to patients in their daily management tasks and those who participated in decision making. Though HF caregiver-related issues are similar to those seen in other chronic illnesses, HF caregivers face unique and specific challenges that increase stressors, such as frequent exacerbations, re-hospitalizations, constantly monitoring symptoms and signs, advances in patients’ therapies (implanted cardiac devices), and patients’ cognitive impairment, with a prevalence rate of 25% to 50% [22].

HF caregivers of male patients had worse physical QoL. Men with HF have inadequate self-care outcomes, are dissatisfied with their health [31], and, moreover, they are four times more likely to be at risk for suboptimal self-care compared to women [32]. As a general rule, males tend to ineffectively use health care services and utilize medical assistance less often than their female counterparts. Additionally, they may perceive themselves as weak or vulnerable when seeking help or advice [32]. Such factors impose unrecognized burdens on their partners. Moreover, worse mental QoL was observed in HF caregivers of patients performing household tasks. The extent to which HF patients perform domestic duties depends on their living conditions, disease severity, and physical or mental limitations. HF caregivers of patients who perform domestic duties may experience high vigilance behaviors to prevent the loved person from debilitation. Over time, constant and high vigilance may lead to fatigue and activate biological processes (e.g., the hypothalamic–pituitary–adrenal axis) associated with poor health. An additive contributor to low mental health is the ongoing effort to maintain a compassionate attitude towards the HF patient. More comprehensively, family caregivers spend a considerable amount of patience on maintaining compassion, empathy, and kindness in their attempt to provide caregiving. The struggle to maintain compassion is a risk factor for developing adverse outcomes, such as depression, decreased QoL, and increased caregiver burden [33].

Surprisingly, patients’ NYHA class was not associated with caregivers’ QoL. One proposed explanation is that the caregivers delivered care at home to stable HF patients and may not be overburdened by caregiving. However, higher caregiver strain is associated with worse patient symptoms (increase in NYHA class). Of note, research focusing on the burden of HF caregivers and how it relates to disease severity is limited [4].

One confounding factor related to the patients’ moderate levels of self-care observed might be their age, since self-care capabilities tend to decline with age [34].

Last but not least, in the present study, the percentage of female participants was higher compared to males, which may have caused a bias in the results, the reason being that women differ from men in their response to stressors. Consequently, anxiety/depression and QoL may have been overestimated. One solution to minimize this bias is to enroll a larger sample size, including participants of both genders with similar socio-demographic characteristics and care demands.

Given that, across OECD countries, one in ten individuals provides support and care to a relative and two-thirds of them are female [35], it is easily comprehended that data exploring gender differences in the caregiving role are limited. Historically, women have had a central role in home caregiving and assumed more responsibilities for domestic tasks, whereas men have had less involvement in home duties, mainly due to spending more time at work or being unfamiliar with caregiving tasks [36]. Although, nowadays, the number of women on the labor market is steadily increasing, women in Greek society still tend to dedicate plenty of time to family responsibilities. Therefore, the current finding may be crucial for developing policies and support interventions considering the role of gender in informal caregiving and focusing on caregiving motivation, training, and emotional burden. Equity in HF caregiving roles and provision of high quality of care demand addressing gender disparities.

### Limitations of the Study

Interpretation of the results should be made with caution, as this study is limited by its cross-sectional design, which does not provide evidence of causal relationships between all dimensions under evaluation. The method of convenience sampling is not representative of all HF caregivers in Greece, thus limiting the generalizability of the results. In the present cross-sectional study, caregivers and HF patients who visited clinical settings for regular monitoring and follow-up care were enrolled, which may reflect a more motivated population. Furthermore, there was no follow-up measurement to permit exploration of all potential changes in QoL and HADs. Moreover, variables regarding caregiving burden, such as length of time delivering care, perceived social support, and patients’ cognitive impairment, were not included. In addition, anxiety and depression were assessed using self-report instruments and there was no information of an established clinical diagnosis provided by medical records or a psychiatric evaluation. One more potential issue that impacts the study’s results is that comorbidities were not recorded (in caregivers and patients); comorbidities imply additional levels of care complexity. As previously articulated, the diversity of HF caregivers’ characteristics was low, since most of them were female and were the patients’ spouses, and as a consequence, the results are not generalizable to males. The sample size was relatively small but significant associations were found. Lastly and importantly, in the current study, only English-language publications were included, and studies in other languages may have been missed.

## 5. Conclusions

Caregivers’ QoL was associated with their anxiety and depression but not with patients’ self-care, anxiety, and depression. In terms of caregivers, factors affecting their physical and mental QoL were as follows: the type of relationship to the patient, gender, age, residency, number of children, level of information on the self-care of the patients they care for, need for learning new skills, provision of support to perform daily management tasks, neglect of their own health, and participation in medical decisions. In terms of patients, the only factor associated with caregivers’ physical QoL was the male gender, and the only factor associated with caregivers’ mental QoL was performance of household tasks.

## 6. Future Perspectives

It is widely known that health care professionals focus on HF patients and often do not assess issues related to caregivers’ health and QoL. Indeed, information regarding the QoL and mental health of informal caregivers still remains limited in the cardiac literature. Characteristics of HF caregivers are a valuable tool to better understand and support this vulnerable population. This information is important for developing interventions (education, emotional support, home care) that alleviate caregivers’ anxiety and depression and promote QoL. Therefore, data about HF caregivers need to be expanded and accurate. For instance, the person providing care (spouse, children) needs to be assessed first; afterwards, the type of care (hours per day, type of tasks) should be assessed, and finally the cost of the care for the caregiver in terms of personal, professional, and social dimensions. Each meeting of health care professionals with HF patients is an opportunity to address caregivers’ needs and health. Equally important for HF caregivers is the support provided by formal health care services within the health care system of each country.

Given the aging of the population, the number of HF patients and their caregivers is expected to increase in the near future. Caregivers are an underrepresented group and future research efforts need to encourage their participation in order to better evaluate their needs. Development of widely accepted specific tools to screen HF caregivers who are at high risk for emotional disturbance is required. This measure would pave the way for future collaborations between countries on a global scale.

QoL and anxiety/depression in caregivers is an area meriting further research. Only when caregivers are free from anxiety/depression and have an optimal QoL will they be able to manage stable chronic HF patients living in the community effectively. In this case, the benefit might be triple: (i) diminished health care costs, since hospital resources would be used to treat more severe or acute HF; (ii) delay of HF progression or decompensation; and (iii) management of the disease within a familiar environment, with the patient’s perception of living a normal life at home, despite their symptoms and limitations.

## Figures and Tables

**Table 1:** Description of caregiver sample (*n* = 110).

	*n* (%)
**Relation to patient**	
Child	45 (40%)
Spouse	65 (60%)
**Gender**	
Male	31 (28.2%)
Female	79 (71.8%)
**Age (years)**	
<40	21 (19.1%)
41–50	24 (21.8%)
51–60	30 (27.3%)
>60	35 (31.8%)
**Education**	
Primary	26 (23.6%)
Secondary	39 (35.5%)
University	37 (33.6%)
MSc or PhD	8 (7.3%)
**Occupation**	
Employed	63 (57.3%)
Household	21 (19.0%)
Pensioner	26 (23.7%)
**Residency**	
Attica	64 (58.2%)
Prefecture capital	24 (21.8%)
Province	22 (20.0%)
**Νο of Children**	
0	31 (28.2%)
1	24 (21.8%)
2	40 (36.4%)
>2	15 (13.6%)
**Need for written HF information** (yes)	95 (87.2%)
**Are you informed about the patient’s self-care**	
Very informed	48 (43.6%)
Informed enough	41 (37.3%)
A little informed	21 (19.1%)
Not informed at all	0 (0.0%)
**Do you support the patient in daily management tasks?**	
Always	42 (38.2%)
Often	38 (34.5%)
Rarely	24 (21.8%)
Never	6 (5.5%)
**Do you neglect your own health?**	
Always	28 (25.5%)
Often	44 (40.0%)
Rarely	26 (23.6%)
Never	12 (10.9%)
**Do you participate in medical decision making?**	
Always	28 (25.5%)
Often	61 (55.5%)
Rarely	17 (15.5%)
Never	4 (3.5%)
**Need to learn new skills?** (yes)	81 (75.0%)

**Table 2:** Description of patient sample (*n* = 110).

	*n* (%)
**Gender**	
Male	73 (66.4%)
Female	37 (33.6%)
**Age (years)**	
41–50	6 (5.4%)
51–60	15 (13.6%)
61–70	28 (25.5%)
>70	61 (55.5%)
**NYHA**	
II	21 (19.1%)
III	48 (43.6%)
IV	41 (37.3%)
**Education**	
Primary	56 (50.9%)
Secondary	36 (32%)
University	14 (12.7%)
MSc or PhD	4 (3.6%)
**Occupation**	
Employed	14 (12.7%)
Household	27 (24.5%)
Pensioner	69 (62.8%)
**Residency**	
Attica	60 (54.5%)
Prefecture capital	36 (32.7%)
Province	14 (12.7%)
**Need for written HF information** (yes)	78 (70.9%)
**Need to learn new skills?** (yes)	53 (58.2%)

**Table 3:** Caregiver QoL (*n* = 110).

	Mean (SD)	Median (IQR)	Cronbach’s a
Physical functioning (Range: 0–100)	19.13 (8.40)	18.0 (10.0–26.0)	0.789
Role-physical (Range: 0–100)	60.00 (40.81)	75.0 (25.0–100.0)	0.850
Role-emotional (Range: 0–100)	56.97 (39.45)	66.7 (33.3–100.0)	0.718
Energy/fatigue (Range: 0–100)	53.76 (24.31)	55.0 (35.0–75.0)	0.893
Emotional well-being (Range: 0–100)	58.65 (23.76)	64.0 (36.0–80.0)	0.923
Social functioning (Range: 0–100)	57.50 (31.00)	56.3 (25.0–87.5)	0.883
Pain (Range: 0–100)	61.93 (31.62)	67.5 (32.5–90.0)	0.913
General health (Range: 0–100)	54.61 (21.75)	62.0 (37.0–72.0)	0.900
Physical Component Summary (PCS)	35.25 (6.48)	35.9 (30.1–40.1)	
Mental Component Summary (MCS)	46.40 (14.52)	47.5 (33.6–59.4)	

SD: standard deviation. IQR: interquartile range.

**Table 4:** Description of anxiety/depression among patients and their caregivers.

	Patients *n* (%)	Caregivers *n* (%)	Cronbach’s a (Patients/Caregivers)
**Anxiety**			0.868 (patients) 0.830 (caregivers)
No (Score ≤ 8)	48 (43.6%)	39 (35.5%)	
Yes (Score > 8)	62 (56.4%)	71 (64.5%)	
**Depression**			0.857 (patients) 0.897 (caregivers)
No (Score ≤ 8)	45 (40.9%)	64 (58.2%)	
Yes (Score > 8)	65 (59.1%)	46 (41.8%)	

**Table 5:** Patient’s self-care behavior (*n* = 110).

	Mean (SD)	Median (IQR)	Cronbach’s a
**Patient Self-Care Behavior** **(Range 9–45)**	19.57 (6.95)	17.5 (14.0–23.0)	0.775

SD: standard deviation. IQR: interquartile range.

**Table 6:** Caregiver characteristics associated with their QoL.

	Caregiver Physical Component Summary (SF36-PCS)			Caregiver Mental Component Summary (SF36-MCS)		
Caregiver’s Characteristics	Mean	Median	*p*-Value ^#^	Mean	Median	*p*-Value ^#^
	(SD)	(IQR)		(SD)	(IQR)	
**Relation to patient**			0.001 *			0.003 *
Child	38.29 (6.36)	39.0 (37.0–41.1)		51.19 (13.74)	54.8 (41.1–63.4)	
Spouse	32.91 (5.72)	33.5 (29.0–35.8)		41.89 (14.38)	38.8 (29.6–54.2)	
**Gender**			0.001 *			0.012 *
Male	38.49 (5.85)	40.1 (36.2–41.3)		51.66 (14.41)	56.7 (43.7–63.4)	
Female	33.96 (6.30)	34.3 (28.9–38.7)		44.31 (14.11)	44.1 (32.6–55.5)	
**Age (years)**			0.001 *			0.001 *
≤40	37.59 (6.89)	39.0 (38.3–41.1)		56.43 (13.98)	63.6 (45.2–65.3)	
41–50	39.22 (6.07)	40.3 (35.6–42.0)		48.54 (12.49)	49.3 (42.4–56.1)	
51–60	35.89 (4.48)	36.2 (31.8–38.6)		47.76 (12.72)	51.8 (37.3–59.0)	
>60	30.21 (4.89)	29.3 (26.3–33.7)		36.91 (12.52)	32.8 (28.4–43.9)	
**Education**			0.061			0.054
Primary	31.59 (5.57)	32.8 (29.1–34.4)		36.51 (12.03)	36.3 (25.4–38.9)	
Secondary	35.86 (6.22)	36.2 (30.6–39.8)		40.81 (13.91)	41.2 (32.6–56.1)	
University/MSc or PhD	37.77 (5.30)	38.9 (35.5–41.1)		43.97 (11.41)	45.5 (40.8–64.2)	
**Occupation**			0.062			0.059
Household	32.75 (5.96)	33.5 (28.4–37.4)		44.50 (14.35)	38.9 (32.6–58.2)	
Employed	35.59 (5.81)	35.7 (33.6–41.0)		47.09 (13.47)	48.9 (41.1–61.6)	
Pensioner	30.81 (6.09)	29.0 (26.8–33.7)		39.94 (13.50)	38.6 (25.4–42.8)	
**Residency**			0.004 *			0.010 *
Attica	36.67 (6.02)	37.9 (32.4–40.4)		49.95 (13.58)	51.8 (40.0–62.3)	
Prefecture capital	35.08 (6.72)	35.9 (29.8–39.8)		42.27 (14.33)	38.6 (31.7–55.8)	
Province	31.40 (6.31)	30.4 (26.3–35.9)		40.61 (15.14)	35.1 (28.4–53.9)	
**Νο of Children**			0.037 *			0.002 *
0	37.72 (6.68)	39.0 (36.2–41.0)		53.22 (15.17)	61.8 (43.7–64.8)	
1	35.11 (5.99)	35.3 (30.7–39.1)		48.43 (13.97)	53.3 (38.6–58.7)	
2	34.15 (6.68)	34.9 (29.0–39.8)		41.52 (12.87)	38.9 (30.8–53.4)	
>2	33.39 (5.37)	32.1 (28.8–36.2)		42.04 (13.16)	40.8 (32.3–53.5)	
**Informed about** **patient’s self-care**			0.001 *			0.001 *
Very informed	37.65 (5.88)	38.9 (35.5–41.1)		52.56 (13.61)	56.7 (44.9–64.2)	
Informed enough	34.68 (5.83)	34.6 (30.2–39.0)		44.75 (13.53)	45.2 (35.2–55.5)	
A little informed	30.98 (6.80)	28.9 (26.1–34.4)		35.82 (11.54)	32.3 (28.4–43.3)	
**Need for written info about HF**			0.107			0.067
Yes	34.88 (6.55)	35.7 (29.3–39.9)		45.56 (14.26)	46.2 (32.8–58.2)	
No	37.73 (5.60)	39.0 (34.6–40.7)		52.10 (15.47)	56.3 (40.0–64.8)	
**Need for learning** **new skills?**			0.005 *			0.001 *
Yes	34.32 (6.58)	34.6 (29.0–39.0)		42.81 (13.63)	43.1 (32.3–54.2)	
No	38.06 (5.37)	39.3 (37.0–40.7)		57.30 (11.50)	62.3 (52.8–65.4)	
**Do you support the patient in daily management tasks?**			0.001 *			0.001 *
Always	32.54 (6.89)	30.7 (26.8–36.7)		36.81 (12.92)	33.9 (25.1–48.1)	
Often	34.99 (5.65)	35.9 (31.4–38.9)		47.89 (12.04)	50.8 (37.2–57.3)	
Rarely	39.35 (4.65)	40.1 (38.5–41.1)		57.98 (9.69)	62.0 (48.3–65.5)	
**Do you neglect your own health?**			0.001 *			0.001 *
Always	31.88 (7.03)	31.2 (26.3–35.8)		36.67 (12.92)	32.6 (25.3–50.3)	
Often	35.60 (5.57)	36.2 (30.6–39.8)		45.00 (12.36)	45.2 (35.9–54.9)	
Rarely	37.32 (6.19)	38.8 (34.2–40.7)		55.14 (12.94)	61.7 (50.2–64.7)	
**Do you participate in medical decision making?**			0.001 *			0.001 *
Always	30.96 (6.96)	29.1 (26.3–34.5)		35.14 (12.90)	31.9 (25.0–39.4)	
Often	36.83 (5.34)	38.6 (33.5–40.3)		49.00 (12.96)	50.7 (39.1–58.7)	
Rarely	36.44 (6.56)	37.1 (31.6–40.4)		53.97 (12.62)	59.5 (43.3–63.3)	
**Caregiver HADs: anxiety**			0.001 *			0.001 *
No (Score ≤ 8)	38.3 (3.2)	38.9 (36.7–40.4)		58.9 (7.0)	59.8 (54.5–64.8)	
Yes (Score > 8)	33.6 (7.2)	32.0 (28.4–39.0)		39.7 (13.0)	37.3 (29.9–48.3)	
**Caregiver HADs: depression**			0.001 *			0.001 *
No (Score ≤ 8)	38.5 (5.2)	39.0 (36.7–41.1)		54.6 (11.2)	57.3 (47.7–63.9)	
Yes (Score > 8)	30.8 (5.2)	30.6 (26.6–34.6)		35.1 (10.4)	33.2 (26.3–41.1)	

* statistically significant (*p* < 0.05). ^#^ calculated by Mann–Whitney or Kruskal–Wallis test.

**Table 7:** Patient characteristics associated with their caregivers’ QoL.

	Caregiver Physical Component Summary (SF36-PCS)			Caregiver Mental Component Summary (SF36-MCS)		
Patient’s Characteristic	Mean	Median	*p*-Value ^#^	Mean	Median	*p*-Value ^#^
	(SD)	(IQR)		(SD)	(IQR)	
**Gender**			0.035 *			0.875
Male	34.5 (6.2)	35.6 (29.3–38.9)		46.5 (15.0)	49.1 (32.6–60.5)	
Female	36.7 (6.8)	39.8 (30.6–41.3)		46.2 (13.7)	45.8 (36.3–57.4)	
**Age**			0.128			0.659
≤60	35.2 (6.6)	35.7 (29.4–40.7)		45.9 (15.8)	52.3 (32.3–57.4)	
61–70	33.5 (5.7)	32.9 (28.9–37.2)		44.8 (12.5)	45.0 (36.5–54.5)	
>70	36.1 (6.7)	37.4 (30.6–40.3)		47.3 (15.1)	47.9 (35.1–61.7)	
**Education**			0.670			0.801
Primary	35.1 (6.9)	36.1 (28.9–40.1)		45.7 (15.2)	46.5 (31.7–59.8)	
Secondary	35.8 (6.7)	37.1 (30.5–41.0)		47.8 (13.8)	50.5 (36.3–61.6)	
University/MSc/PhD	34.6 (4.8)	34.8 (30.8–38.2)		45.9 (14.2)	50.3 (33.6–56.1)	
**Occupation**			0.089			0.029 *
Employed	35.1 (5.4)	35.7 (30.7–39.0)		49.1 (13.6)	52.8 (37.3–59.4)	
Pensioner	36.0 (6.8)	37.1 (31.1–40.3)		47.1 (14.6)	47.6 (35.1–60.8)	
Household	31.3 (5.8)	29.1 (26.8–36.0)		36.1 (12.5)	34.1 (26.3–44.0)	
**Residency**			0.67			0.801
Attica	35.1 (6.9)	36.1 (28.9–40.1)		45.7 (15.2)	46.5 (31.7–59.8)	
Prefecture capital	35.8 (6.7)	37.1 (30.5–41.0)		47.8 (13.8)	50.5 (36.3–61.6)	
Province	34.6 (4.8)	34.8 (30.8–38.2)		45.9 (14.2)	50.3 (33.6–56.1)	
**NYHA**			0.433			0.138
I-II	34.6 (6.3)	34.6 (29.0–39.0)		47.1 (13.1)	44.9 (36.1–55.5)	
III	36.4 (6.5)	37.1 (30.7–40.3)		49.2 (13.5)	51.2 (36.8–60.9)	
IV	34.2 (6.5)	35.6 (28.4–39.6)		42.7 (15.9)	41.9 (25.7–57.1)	
**Need for written information**			0.752			0.200
Yes	35.2 (6.6)	35.5 (30.2–40.1)		45.3 (13.9)	46.2 (33.6–56.7)	
No	35.3 (6.3)	38.2 (29.2–40.1)		49.0 (15.8)	53.7 (35.5–63.2)	
**Need for learning new skills**			0.100			0.705
Yes	34.1 (6.1)	34.9 (28.9–39.0)		46.9 (15.0)	48.3 (32.6–61.6)	
No	36.4 (6.7)	37.2 (30.6–40.3)		45.9 (14.1)	46.7 (35.7–58.3)	
**Patient HADs:** **anxiety**			0.985			0.453
No (Score ≤ 8)	35.4 (6.5)	35.6 (30.4–39.2)		47.8 (13.1)	47.6 (35.6–60.3)	
Yes (Score > 8)	35.1 (6.5)	36.2 (29.4–40.3)		45.3 (15.5)	46.2 (32.3–59.4)	
**Patient HADs: depression**			0.502			0.059
No (Score ≤ 8)	34.9 (6.0)	34.9 (29.3–39.0)		49.6 (13.5)	54.1 (37.2–62.2)	
Yes (Score > 8)	35.5 (6.8)	36.2 (30.4–40.3)		44.1 (14.9)	43.8 (31.9–56.4)	
		**Spearman’s Rho**			**Spearman’s Rho**	
**Patient self-care behavior**		−0.152	0.116		−0.072	0.459

* statistically significant (*p* < 0.05). ^#^ calculated by Mann–Whitney or Kruskal–Wallis test.

**Table 8:** Impact of factors on caregiver’s QoL.

	Caregiver Physical Component Summary (SF36-PCS) ^#^		Caregiver Mental Component Summary (SF36-MCS) ^#^	
	b coef (95% CI)	*p*-Value	b coef (95% CI)	*p*-Value
**Caregiver (Spouse vs. Child)**	−0.87 (−4.31–2.58)	0.617	2.04 (−4.36–8.43)	0.526
**Caregiver Gender (Female vs. Male)**	−1.79 (−4.78–1.19)	0.235	−4.07 (−9.59–1.45)	0.146
**Caregiver Age**				
≤40	Ref		Ref	
41–50	2.94 (−0.87–6.74)	0.128	−5.92 (−12.65–0.81)	0.084
51–60	−1.54 (−5.85–2.77)	0.478	−4.22 (−11.73–3.29)	0.265
>60	−3.39 (−8.69–1.92)	0.207	−7.96 (−17.35–1.43)	0.095
**Caregiver Residency**				
Attica	Ref		ref	
Prefecture capital	1.15 (−2.19–4.50)	0.492	−3.29 (−9.06–2.47)	0.257
Province	1.33 (−2.31–4.98)	0.468	1.64 (−4.41–7.69)	0.59
**Caregiver Νο of Children**				
0	Ref		ref	
1	2.91 (−1.91–7.73)	0.232	3.67 (−4.57–11.90)	0.377
2	3.17 (−0.81–7.15)	0.116	2.72 (−4.34–9.78)	0.443
>2	4.49 (−0.59–9.58)	0.082	3.52 (−5.35–12.39)	0.431
**Caregiver is “informed about patient’s self-care”**				
Very informed	Ref		ref	
Informed enough	−0.66 (−3.86–2.54)	0.683	−0.22 (−5.84–5.39)	0.937
A little informed	1.56 (−2.93–6.06)	0.489	0.71 (−7.11–8.52)	0.857
**Caregiver “needs to learn new skills” (no vs. yes)**	0.90 (−2.72–4.53)	0.621	−0.62 (−6.83–5.59)	0.842
**Caregiver “supports patients in daily management tasks”**				
Always	Ref		ref	
Often	−0.05 (−3.35–3.24)	0.974	4.25 (−1.93–10.44)	0.174
Rarely	2.66 (−1.39–6.71)	0.195	7.90 (0.49–15.30)	0.037 *
**Caregiver answers to “Do you neglect your own health?”**				
Always	Ref		ref	
Often	−0.09 (−3.64–3.45)	0.958	−4.62 (−12.91–3.66)	0.269
Rarely	−1.22 (−5.32–2.88)	0.554	−4.11 (−13.76–5.54)	0.397
**Caregiver “participates in medical decision making”**				
Always	-		ref	
Often	-		12.19 (3.23–21.14)	0.009 *
Rarely	-		14.23 (4.15–24.31)	0.006 *
**Patient** **Occupation**				
Employed	-		ref	
Pensioner	-		−4.07 (−9.72–1.58)	0.155
Household	-		−7.17 (−15.27–0.94)	0.082
**Caregiver HADs: Anxiety (yes vs. no)**	0.59 (−2.58–3.77)	0.71	−9.27 (−14.87–3.68)	0.002 *
**Caregiver HADs: Depress. (yes vs. no)**	−6.22 (−9.70-−2.73)	0.001 *	−8.21 (−14.85–1.57)	0.016 *

b coef: b regression coefficient. CI: confidence interval. ref: reference category. * statistically significant (*p* < 0.05). ^#^ the model includes acceptable factors after multicollinearity assessment.

## Data Availability

The original contributions presented in this study are included in the article. Further inquiries can be directed to the corresponding author.
